# A qualitative exploration of how job insecurity influences the worker roles of youth that are homeless

**DOI:** 10.1177/10519815261421500

**Published:** 2026-04-02

**Authors:** Mogammad Shaheed Soeker, Alo Helene Bester, Anelisa Mvubu, Grete Boonzaaier, Nelma Roodman, Marili Beukman, Monzenick Fourie

**Affiliations:** 1 Occupational Therapy Department, University of the Western Cape, Cape Town, South Africa

**Keywords:** work, unemployment, vocational rehabilitation, qualitative research, poverty

## Abstract

**Background:**

In South Africa, there is an average of 69.6% of unemployed youth between the ages of 15 and 34. There is a lack of research regarding youth who are homeless and their perspective on their worker role.

**Objective:**

The aim of the study is to explore and describe the experiences and perceptions of homeless youth with regard to the influence of job insecurity on their worker roles.

**Methods:**

The study used an exploratory study design using a qualitative approach through in-depth, semi-structured interviews. Purposive sampling methods was used to select six research participants and two key informants. Two semi- structured interviews were conducted with six individuals regarded as youth who are homeless and two key informants.

**Results:**

Four themes emerged from the study: 1) Challenges related to accessing support structures that affect home and job security, 2) A holistic program that focuses on personal and work specific skills creates opportunities for employment. 3) **“**Being able to forgive oneself”, is a start to adaptation to difficult circumstances and 4) Multidimensional support for youth who are homeless to get employed and maintain employment.

**Conclusion:**

The novel finding of this study is that that when youth get support, it significantly increases employment opportunities, which helps the youth who are homeless to adapt to the difficult circumstances they face daily.

## Introduction

Individuals who experience job insecurity have major difficulties that impair their social standing, mental health, and financial security.^1^ Job insecurity can make already difficult situations much worse for vulnerable groups, such as homeless adolescents, by posing considerable obstacles to obtaining secure housing, nutritious food, and medical treatment. With an estimated 4.2 million young people suffering from homelessness in the United States alone each year, homelessness is a growing concern throughout the world.^2^ Whereas in South Africa 55,719 people are homeless per year^3^ (STATSA, 2022). Youth homelessness is considered a societal issue that has a variety of effects on society as a whole.^4^ In South Africa, the average rate of unemployment among youth ages 15–34 is 69.6%.^6^ Additionally, within the context of South Africa other contributing factors of job insecurity and opportunities, include race and gender.^5^ Homeless youth frequently encounter substantial employment hurdles, such as inadequate schooling and unstable housing. If youth who are homeless have work, they may still experience job insecurity due to unstable scheduling, part-time employment, and precarious contracts.^6^ Youth who are homeless take on worker responsibilities influenced by their environment, social networks, and general economic and political circumstances due to job insecurity. Further research is required due to the void in research on the perspectives of youth who are homeless about their worker roles and employment experiences. In South Africa and internationally there is limited research that explores how job insecurity influences work related skills development of homeless youth. The gap in the current research is reflected by a lack of research in South Africa and internationally with regard to the meaning that unemployed youth associate with the worker role particularly when there is a scarcity of work. The current study contribute to this understanding as there are no studies from a South African perspective that explore the views of homeless youth regarding the challenges and facilitatory aspects that assists them in finding and maintaining employment. A rise in job insecurity and increase in homelessness in South Africa, causes youth who are homeless to turn to risky coping techniques such as substance abuse, gangsterism.^7,8^ These actions may lead to less favourable health results. The aim of the current research project was to explore the influence of job instability on the worker role of adolescents who are homeless. Moreover, the study aims to recommend how to enhance income-generating initiatives that individuals who are homeless believe are solutions that would support them.

## Literature review

In the literature review there will be a discussion on the prevalence of unemployment in South Africa, the barriers that inhibit and the enablers that support youth in finding employment

### Unemployment statistics of youth in the South African and international context

According to literature, unemployment among youth is a continuous issue prevalent in South Africa.^5^ Various factors influence unemployment for youth who are homeless, namely the size of the youth labour force, lack of work demand, and lack of experience compared to adults.^5^ Research suggests that even youth who are educated struggle to find work due to these same factors.^8^ Statistics prove that there is an average of 69.6% of unemployed youth between the ages of 15 and 34 in South Africa.^9^ Statistics indicate factors in South Africa contributing to difficulties in securing job and opportunities to find employment include race and gender.^5^ In South Africa a driver for homelessness is historical disadvantage, which includes apartheid, where the African population was segregated. During apartheid, various government mechanisms drove people to homelessness, which included forced removals, denial of the use of apartheid type of documents such as the “dompas”, and legislated landlessness.^10^ Job insecurity has been described as the perceived fear of losing one's current job for unexpected and uncontrollable events.^11^ Whereas job instability is described from an organizational context as an event or practice involving the reduction of workforce in the organisation, thus influencing the creation or the availability of work or employment opportunities for society.^12^ It could be argued that unemployment has a strong link with job instability and job insecurity. It could therefore also be argued that limited work opportunities cause youth to struggle to find and maintain employment, thus reinforcing both job insecurity and job instability. The latter concepts continue to limit youth who may have limited work skills, thus contributing to unemployment.

According to Vázquez,^13^ health issues, substance abuse, disability, and criminal records are all contributing factors to youth unemployment. Furthermore, stigma often negatively influence the emotional well-being of individuals within society who are homeless, as well as their ability to gain support and assistance.^13,14^

To overcome and address barriers, supportive employment can be an option to help homeless people return to work.^15^ There is an important relationship between employment and housing, and the relationship is bidirectional.^15^ Thus, employment facilitates the ability to maintain housing, and housing in turn facilitates maintaining employment. Several studies suggest that supported employment services and supported housing are extremely important to reduce homelessness. The biggest barrier that existed before homelessness was substance use.^15^ Positive outcomes of homelessness include adapting strengths and attitudes, this contribute the argument that homelessness does not only have negative outcomes. Homeless people often attend job finding services that will prepare them for interviews and difficult questions by having them participate in mock interviews for practice.^15^

### Employers’ expectations regarding employing youth

In a research study conducted by Pehin Dato Musa and Pg Hj Idris,^16^ they state that youth are experiencing increasing uncertainty when transitioning into the labour market. A leading cause of this is that youth prefer a job that provides them with a stable income rather than doing enterprising work with a lot of uncertainty. Additionally, youth are seeking employment in governmental jobs, and not within the private sector due to the security and privileges that government jobs provide.^16^ Migration plays a role in youth getting employed, as Ohlmann^17^ highlighted within the rural areas of South Africa, employment opportunities are scarce and often young individuals are faced with the decision to relocate to a larger area with more opportunities.

From the above literature that has been reviewed it can be argued that although there are various challenges identified that influence the worker role of individuals who are homeless, there are minimal research that focus on the how supportive factors in the environment influences the worker roles of individuals who are homeless.

## Aim and objectives

The aim of the study is to explore and describe the experiences and perceptions of homeless youth with regard to the influence of job insecurity.

### Objectives:

- To explore and describe the experiences and perceptions of homeless youth about the challenges that they experience in finding and maintaining employment.
- To explore and describe the experiences and perceptions of homeless youth about the enablers/facilitators that they experience in finding and maintaining employment.
- To explore and describe the experiences and perceptions of homeless youth about how they adapt to their worker role.
- To explore and describe the experiences and perceptions of homeless youth about suggestions to enhance income generating programs.

### Methods

The researchers used a qualitative exploratory research design, as the study aims to interpret and critically understand people's experiences around homelessness and their participation in the occupation of work. Exploratory research is used to investigate a broad concept or a particular subject when there is no prior information or paradigm to use.^18^ It guides a particular study to obtain information on a new question or topic not researched before.^19^ The researchers completed the research study by following the methodological steps as described in the sections that follow.

### Participant recruitment and sampling strategy

The research study was conducted at the [Name of the NGO], which is a registered non-profit organisation that provides a temporary home, informal employment, and skills development for the homeless. The researchers have had no previous interaction or collaboration with the above mentioned NGO. Purposive sampling was used in the current study, it is also known as judgmental sampling, which means that the sample has been selected based on the knowledge that the research participants may have regarding the research area of concern.^20^ The participants have therefore been selected based on a set of inclusion and exclusion criteria. Inclusion criteria included youth who are homeless with at least six months of prior work experience, between the ages of 18 and 35, who were able to communicate in isiXhosa or Afrikaans or English. Exclusion criteria included individuals with uncontrolled psychiatric symptoms that may affect their ability to participate in the study. In the current study individuals who were not be able to communicate or respond due to displaying acute psychiatric symptoms were excluded from the study. Due to the nature of qualitative research the research participants selected six individuals that best met the inclusion and exclusion criteria of the study. The two key informants were selected based on their experience in particularly working with youth that are homeless. Key informants in the current study included people working at non-governmental organizations that work with youth who are homeless. The key informants were chosen as they would be able to provide an understanding of how the programs that are available, help youth who are home to find employment. The views of the key informants would also enhance and triangulate with the views obtained from the youth who were unemployed. The researchers selected six participants and two key informants. Of the six participants, four of the youth who were homeless were male and two female. According to Cresswell,^21^ he argues that in qualitative research, the number of interviews could range from 05 to 25 interviews in total. In the current study one interview was conducted with each research participant, before data saturation was achieved.

### Data collection techniques

The data collection methods for the current study included the use of semi-structured interviews. These are interviews that consist of several key or open-ended questions that help to define the areas to be explored, whilst also allowing the interviewer to diverge to pursue an idea or response in more detail.^22^ The researchers conducted one semi-structured interview individually with each participant. One additional member checking interview was conducted with the research participants. The researchers were not familiar with the research participants nor with the personal contexts of the research participants. Research bias was mitigated by the use of a reflexive journal as advocated by Krefting,^23^ in this journal any pre conceived ideas that the researchers had were documented. In total, 8 interviews (these interviews consisted of 1 interview per research participant). The duration of the interviews were 30 to 50 min. All the research participants agreed with the findings of the study. The interviews were conducted in a quiet consultation room of the NGO that provided support services to the homeless. The researcher provided the research participants with an opportunity to ask any questions that they may have had before they consented to participate in the study. The interviews were audio recorded before it was analysed. Data analysis will be described in the next section of the article.

### Data analysis

The data of the current study were analysed using thematic analysis. The data was analysed manually using the techniques as advocated by Tesch in the study.^24^ This process was followed by generating initial codes manually in all eight interviews. The codes were then sub-categorized, categorized and grouped under four different themes that highlight the study focus area (examples of codes included terms such as unemployment and skills development). In the current study all the participants responded to the interviews in English, therefore none of the quotes required interpretation. To ensure the rigour of the processes followed in the study, the researchers used the concept of trustworthiness.^23^ This process consists of a combination of strategies that ensures the quality of the research data, namely, credibility, transferability, dependability and confirmability.^23^

### Ethics procedures that were followed

Permission to conduct the study was obtained from the NGO in the area of where the research was conducted. The researchers described the study in detail to the directors of the organization and requested that they inform possible research participants about the research study. The NGO were informed that that they could ask any questions related to study and that a copy of the research report would be made available to them at their request. The involvement of the NGO as a gate keeper in the current project did not affect the response of the research participants. The research participants were also informed that the findings of the study would be disseminated after the completion of the research project. Information about the study was given to the interested participants in the form of an information letter prior to data collection, along with consent forms, to ensure that the principle of informed consent was adhered to. Obtaining this informed consent minimized the risk of harm to participants and therefore achieved non-maleficence for this study. As the research participants were regarded as a vulnerable group, the researchers were very sensitive to ethics related procedures as it relates to managing vulnerable populations in research. The researchers requested that the NGO provide information pertaining to the study to the research participants (youth who were homeless) who in turn contacted the researchers if they were interested in participating in the study. To ensure confidentiality and anonymity, the researchers did not use the participants’ names in the study but rather unique numbers as a form of identification. The research participants were informed that they could stop participating in the study at any time. The researchers did not provide any incentives to the research participants as the interviews were conducted on the premises of the NGO that provided skills training to the research participants. The researchers had the contact details of health- related professionals available should the research participants require it due to the nature of the research study. The current study was approved by the Institutional Review Board of the [Name of the University] (Ethics number: BM23/4/15).

## Results

Table 1 describes the themes and categories. Table 2 describes the demographics of the research participants of the study. In the sections below the researchers describes the themes that relate to the objectives of the study. Theme 1 describe the barriers that the homeless individual experience when finding employment. Theme 2 describes the factors that assist individuals that are homeless in finding and maintaining employment. Theme 3 relates to the process of adapting to their worker roles. Finally theme 4 focuses on suggestions to improve income generating programs to enhance the work related skills of individuals who are homeless.

**Table 1.** Group Themes, Categories and Subcategories Table.

Theme	Category
Theme 1: Challenges related to accessing support structures that affect home and job security.	Category 1: Poor levels of education and qualifications result in job inexperience and unemployment.
	Category 2: The influence of culture and rebellious behaviour on the individual's capacity to be employed.
	Category 3: The employment opportunities are limited due to addiction and prior legal offences.
	Category 4: The stigma projected by communities towards homeless youth negatively influences finding employment.
Theme 2: A holistic program that focuses on personal and work-specific skills creates opportunities for employment.	Category 1: The program facilitates community reintegration.
	Category 2: Programs that are occupation-based enhance one's worker role.
Theme 3: “Being able to forgive oneself” is a start to adaptation to difficult circumstances.	Category 1: Internal and external support mechanisms can help one adapt to difficult circumstances.
	Category 2: Being able to build positive relationships with people.
Theme 4: Multi-dimensional support for homeless youth to get employed and maintain employment.	Category 1: Increase job opportunities through skill enhancement, resulting in the maintenance of employment.
	Category 2: Inclusion of supportive elements in existing rehabilitation programs to facilitate the resumption of the worker role.
	Category 3: A focus on self-growth and gaining acceptance in the workplace.

**Table 2.** Table of demographics of research participants.

Name of participant	Age	Gender	Education level	Employment status
P1	25	Male	Grade 12	General worker (Recycling Industry)
P2	23	Female	Grade 11	General worker (Recycling Industry)
P3	30	Male	Grade 12	Unemployed
P4	27	Female	Grade 11	Unemployed
P5	33	Male	Grade 11	Unemployed
P6	35	Male	Grade 10	General worker
KEY INFORMANTS	Age		Qualification	Employment status
K1	35	Female	Social Worker	(NGO)
K2	40	Male	No formal qualification	(NGO)

### Theme 1: Challenges related to accessing support structures that affect home and job security

#### Category 1.1. Poor level of education and qualifications resulting in poor job experience and unemployment

Participant one (P1) shared that she struggled to find employment as a result of not having appropriate documentation, such as an identity document and resident papers, as well as not having a proof of address due to being homeless. The participants indicated that if you are a non-South African resident, then you will struggle to obtain the necessary permits to participate in civic activities such as finding employment. Usually, these permits to reside in South Africa expire within six months and then the individual needs to re-apply for residence. One participant indicated:

“I can say it's papers, resident papers and permits, because most of the jobs they ask you why this instead of your passport, yes your passport and asylum seeker and if your papers expired you must then wait for long time to get another one, it is long processes and that is why I struggle to get a job. Also some jobs I am not qualified to get.. (P1)

Participant one further mentions that sometimes she finds it challenging to find a job because she is not qualified and does not have the necessary work experience. It was clear that lack of adequate work and skills experience, low levels of education and no further qualifications played a big role in the challenges that the youth who are homeless experience in finding and maintaining employment.

#### Category 1.2. Culture and rebellious behaviour influence the individual's capacity to be employed

The category of culture and rebellious behaviour describes some of the barriers to finding employment and this emerged between all the participants and key informants. The key informant said:

“It's big and it works with cultures you see. With the black culture is education and criminal record with the coloured culture. It's mostly education, also with a criminal record and then the worst part is that they have families, they have good homes, the coloured community, but because of the drug abuse and the criminal, the employment is not easy to find.” (KI 1)

The participants further describe rebellious behaviour as an inhibitor and a challenge in finding employment. The participants emphasised that dropping out of school, not listening to parents, and engaging in criminal activities all contributed to their struggle in findings employment. One participant said:

“I was still smoking marijuana, and I couldn’t focus because it distracted me if I completed school and listened to my parents” (P3)

#### Category 1.3. Employment opportunities are limited due to addiction and prior legal offence convictions

This category highlights that legal offences, conviction and addiction all contribute to youth who are homeless experiencing a limitation in employment opportunities. The participants described the latter as the main barrier to limiting employment opportunities. The key informant said:

“…so, one biggest one that we have gotten from most employers, people who want to employ, but when it comes to homeless people, they have so many criminal records. Then the white community has five degrees, three degrees, four degrees, but due to the drug abuse, the job is very difficult to do. (KI 1)

Another participant indicated that in addition to having a criminal record, having a history of drug use limits your chance of findings employment as employers are reluctant to employ you. The key informant said:

“but because of drug abuse and the criminal records, the employment is not easy to find.” (KI 1)

Furthermore, one participant reflected on youth who are homeless who was a pharmacist that lost his job due to drug use. The key informant indicated:

“They told him you can come back as long as you leave the drugs. Till today, still on drugs. But they are willing to give them their job.” (KI 1)

#### Category 1.4. The stigma projected by communities towards youth who are homeless negatively influences finding employment

This category of “Stigma Projected by communities” was viewed as a barrier, emphasizing the difficulties that youth who are homeless experience when looking for jobs as a result of the preconceived ideas from society. One of the participants described the stigma as a one of the challenges they experience when looking for work. The participant said:

“You can actually see when you are homeless, they don't want to give you a job.” (P2)

Ma’am, to be honest with you, the people they judge you on your looks, man outside man. If you're going to look for work they think maybe you're going to steal or they want to know what agency or you understand trying to say that that you can't just go and ask job because they always tell you they got someone in the garden or stuff like that. To be honest with you the people that don't give, you a job if you are homeless. (P1)

### Theme 2: A holistic program that focuses on personal and work specific skills creates opportunities for employment

#### Category 1: Program facilitates community reintegration

The first category for facilitators is that a holistic program facilitates the community reintegration of youth that experience homelessness. Aspects such as the facilitation of second-phase housing and enabling youth who are homeless to live as a family unit were regarded as enablers to reintegrate youth back into their communities. A participant expressed that to get his life back, he needed a program that provided the space to stay clean and eat. The participant said:

“The program that I was in at SPC, they helped us a lot by staying clean, eating, focusing on our life, getting our life back and that.” (P6)

A key informant explained the importance of guaranteed housing. He stated that their program provided accommodation over a period of six months and further explained that through accommodation, a youth who is homeless could become motivated. The participant said:

“But now accommodation is when we say we have a second phase house where we can give you accommodation for six months, that's guaranteed.” (KI 1)

“The moment they get accommodation, they get motivated.” (KI 1)

Another key informant explained that reconnecting with family and yourself is an important aspect of reintegration into work. Connecting with your family unit and making peace should form part of a holistic approach to optimal community reintegration. The participant said:

“You need to run through a program to find yourself, to reconcile with yourself, and to reconcile with the people to get out here with you here, in the working environment again” (KI 2)

#### Category 2: Programs that are occupation based enhances one's worker role

The youth that attend the programs at the Place of Safety can reside temporarily in a safe space. However, they are required to use “vouchers” in order to use facilities that are available at the safe space, such as receiving a cooked meal, bathing and a safe space to sleep. Participants expressed that having access to facilities to complete their basic activities of daily living (bathing, eating, personal hygiene) assisted them with gaining confidence and keeping them in the right mindset when reintegrating back into the community. The participant said:

“I tried staying in the right mindset. From work, I went directly to the shelter, washed myself, prepared myself for work the next morning,”. (P4)

Shelters can provide the support that youth require in order to increase their self-esteem after they have become homeless, and face various challenges daily. The increase in self-esteem could then improve their lives. The participants said:

“But I mean you need that self-esteem to pull back”. (KI 2)

“The only way we could get the job is to get ourselves into rehabilitation, and then they have to refer us to grow” (P6).

Another participant described the important role that shelters played in helping individuals find improve their work skills:

“ah so, then they said you can stay here and enter into a program. They asked me what I wanted and I told them I want a job and place to stay obviously I was homeless and then they got me here. And they said I must be in this program called GIA. They said, you must be in that program to get a job, in that program you just talk about your struggle and everything and all that stuff that is what we talk about it. And then after six weeks on GIA you get a job” (P1)

### Theme 3: “Being able to forgive oneself”, is a start to adaptation to difficulty circumstances

#### Category 3.1: Internal and external support mechanisms can help one adapt to difficult circumstances

There are many adaptations that youth who are homeless engage in to find and maintain employment. The one key informant said:

“you need to run through a program to find yourself, to reconcile with yourself, and to reconcile with the people to get out here with you, here, in the working environment again.”. (KI 2)

The finding above emphasises the importance of being able to forgive oneself with internal and external support mechanisms. These mechanisms can help one adapt to the difficult circumstances being experienced. When looking at the internal mechanisms, having a positive attitude is fundamental to facilitating change in a person. One participant said:

“I wanted to do something that made me feel good and I could not go back to smoking” (KI 2)

#### Category 3.2: Being able to build positive relationships with people

Looking at external mechanisms, having community support can assist an individual in adapting to the challenges in their life. One participant said:

“Not like (name of shelter) them, to be honest with you not like (shelter). Not like (name of shelter) outside here, ma'am. Then you are gonna get people (from the community) who gonna give you a package (clothes and food) like what I have got on now. I got it in the parking lot on Friday.” (P2)

Although it is understandable that employers are taking a risk by employing youth who are homeless with possible criminal records and substance use histories in their businesses, the community can play a fundamental role in employing these individuals and giving them hope to change their future. Another key informant said:

“Spar (supermarket) is helping a lot now. In training them on how to be how to be packers until workers. So that has been helping. But it's only for those who are out of drugs and who have places, and that's a less number.” (KI 1)

### Theme 4: Multi-dimensional support for youth who are homeless to get employed and maintain employment

#### Category 4.1: Increase job opportunities through skill enhancement resulting in the maintenance of employment

Within this theme, the participants reported that employment opportunities increased when they participated in skills training programs. Some of these skills training programs included opportunities to enhance the participant's computer literacy. A participant said:

“At the beginning, I got to Grow, I attended class. They are calling the class GIA” (P4).

One participant indicated how the participation in a program at a shelter helped them develop a routine and enhance their motivation. He said:

“That is the thing (the shelter) also motivate you. To get up. Because, you know, we don't get up for work. No money and it's basically the same.” (P2)

In addition to the above skills classes, the participants were also provided with money management skills that enhanced their ability to manage their salary and promote financial independence among youth who are homeless.

“What we are trying to help them with is that they can work with, with, with their own finance.” (KI 2)

#### Category 4.2: Inclusion of supportive elements in existing rehabilitation programs to facilitate the resumption of the worker role

Participants indicated that before they could obtain shelter and the services of social workers from Non-Profit Organisations they were encouraged to participate in rehabilitation programs. Participants therefore were no longer homeless, but had a residential shelter as a temporary aid, while attending rehabilitation programs for substance use, where they participated in skills training which eventually led to them finding casual employment. One participant said:

“The job that I have at NGO right now is not a permanent job, but an opportunity” (P4)

The finding above reinforces appreciation from participants regarding all the opportunities they received from the non government organisation. These opportunities ranged from the provision of shelter, safety, skills training, rehabilitation programs, and eventually employment opportunities to further promote independence and facilitate the resumption of their worker role.

#### Category 4.3: A focus on self-growth and gaining acceptance in the workplace

Lastly, it was evident that youth who are homeless do not only need supportive elements and skills training but also the need for personal growth and to gain acceptance in the work environment. This was facilitated through enhancing respect through the role modelling of good behaviour from the employers from the NGO and support through daily motivational sessions. The latter enabled an environment with appropriate behaviour at the NGO's training facility and ultimately the work environment that is being facilitated. Another key informant indicated:

“We forget our morals, and we think the world is against us. So, we lash out on anybody. Anger management, that is one number that they need”. (KI 2)

## Discussion

The purpose of this study was to gain a deeper understanding of the experiences and perceptions of youth who are homeless about the influence of job insecurity on their worker role. This section discusses the barriers and facilitators related to objective one and objective two. Furthermore, the adaptation strategies used by individuals that are homeless and suggestions to enhance income generation programs as they relate to objective three and four are discussed. The novelty of the findings is that in the discussion section that follow the barriers and facilitators are particularly from the perspectives of youth that are homeless from a developing country such as South Africa.

### Barriers

The participants in this study mentioned several barriers to finding and maintaining employment. The barriers were described in detail in theme one of the current study. The first category of barriers was from a personal nature, example, a lack of education and official identification documents and having a criminal record.

According to Huffman et al.^25^ lack of education, lack of identification, are a few examples of barriers to employment. Many participants did not have access to official identification. Due to this lack of education, people are unable to obtain work, that results in employment gaps and additional joblessness. Some participants mentioned difficulty in finding work due to their criminal records. When discussing the difficulties, a second level of barriers was evident in obtaining and maintaining a job, participants mentioned a lack of social support aspects. These included difficulties in living in shelters, unfavourable peer interactions and little support from potential employers. The social career development theory, describes career development by the interaction between self-efficacy, outcome expectations, interests, and the social environment, ultimately influencing an individual's career choices and performance.^26^ In the current study it was evident that the self efficacy beliefs of the research participants were negatively affected as they experienced difficulties in the shelter coupled by a lack of support in the workplace. The perceived lack of acceptance and support from both the community and employers limited their ability to find and maintain employment. The latter being exacerbated by economic factors such as the high unemployment rate in South Africa, which was seen as the main cause of homelessness.^27^ It could therefore be argued that the unemployment rate limits employment opportunities for individuals that are homeless.

Furthermore, becoming homeless is a major life-changing event that causes individuals who are homeless to learn how to adapt to survive.^28^ The participants expressed how their lives have changed drastically as a homeless person and how the various barriers which contribute to homelessness influenced how they had to adapt to their worker roles. This resulted in the participants financially depending on NGOs and various homeless shelters as a means of survival and receiving support.

### Enablers

In theme two, participants expressed that through housing programs, they are reintegrated back into their communities. It was found that guaranteed housing resulted in youth who are homeless to be more motivated and committed to work. Similarly, Slesnick^29^ found that youth who are homeless and have stable housing have a greater chance of being employed. They found that, due to improved housing stability, youth who are homeless turned to legal avenues to generate income.^29^ Other research suggests that through an independent living program, youth who are homeless reported a decrease in substance use, and their self-reported health improved.^30^

In theme two, it was found that through programs, youth who are homeless can reconnect with themselves and with their families, which is an important part of community reintegration. A study found that by addressing a toxic family environment, substance use and family functioning improved.^31^ Others suggest that family cohesion can be improved.^32,33^ Mayock^34^ explained that participants were empowered when they had more contact with their families and the relationship was better. In contrast, Noh^35^ found that family intervention did not affect substance use. The participants highlighted that having access to the shelters enabled them to complete their basic activities of daily living. This improved their psychosocial well-being, as the participants described that completing these essential tasks assisted them with the process of reintegrating back into their communities. As Jayatunge,^36^ stated that when youth live on the streets, they could have hygiene related problems due to their inadequate hygiene practices. Due to their unfortunate life experiences, it can also affect their identity and well-being. Wang, Liu and Deng^37^ further describe that in addition to the latter, an unsupportive social environment may hinder an individual's work identity and career development. Whereas a supportive social environment will facilitate a positive worker identity and positive career development.

The participants have expressed that attending the rehabilitation programs provided them with various opportunities to gain work-related skills to become employed and that it provided them with a chance to gain experience in working in competitive employment. Similarly, Jayatunge,^36^ stated that doing vocational training, and skills development for youth who are homeless can assist with ensuring more financial stability. Furthermore, Jayatunge^36^ states that the vocational training provides individuals who are underprivileged with an increase in security and self-esteem. The above is also evident from the work conducted by Yousaf^38^ that focused on the impact of technical and vocational education and training on alleviating poverty in developing countries. They argue that vocational education reduces poverty at all levels of adult skills, however, it remains significant at low levels.

### Adaptations

The term “occupational adaptation” was used by Schkade and Schultz^33^ to describe an internal and normative process and occupation as a means of adaptation in their model of occupational adaptation. The adaptation strategies that youth who are homeless experience is described in theme three of the current study. According to Schkade and Schultz,^39^ human growth entails an ongoing process of adaptation based on how people respond to varied occupational difficulties. Being able to forgive oneself, is a starting point towards adapting to the difficult circumstances one experiences when being homeless. The theme discusses the importance of internal support systems such as a positive attitude and willingness to change when wanting to adapt to difficult situations. Psychological resilience refers to recovering quickly after facing a traumatic experience.^40^ In a study conducted by Karaırmak et al.,^40^ it was evident that individuals who have psychological resilience can engage better in interpersonal relationships and with better problem-solving skills.

External support can include positive family and community support as well as participation in training and skills training provided by organizations. The literature stated that family, individual and environmental support is what is needed to create psychological resilience for one to be able to forgive themselves.^41^ This support can facilitate adaptation although individuals could experience challenges in building these relationships due to the stigma they experience and the lack of feeling included in society. Babik and Gardner^42^ indicates that this sense of not belonging may negatively affect their confidence levels. Homelessness is a major life-changing event that causes individuals who are homeless to learn how to adapt to survive. To overcome and engage with these challenges the youth who are homeless need to reconcile with themselves and their past behaviour. Studies have confirmed that forgiveness has a positive effect on one's psychological well-being.^43^ And that engaging with self-forgiveness will allow the individual to be more passionate about themselves and allow them to take responsibility for their past rather than experiencing guilt about their past and their mistakes.^44^ This will eventually enable the youth to avoid unnecessary conflict and aim to build healthy work and family relationships in their communities to be employed and maintain employment.^45^

### Support

Theme four found aspects of multi-dimensional support that were important to youth who are homeless to find and maintain employment. When engaging in skills training programs, participants emphasized that youth could experience an increase in employment opportunities. A study done by Slesnick et al.^46^ presented that facilities can be useful for training and provide access for youth to be employed and maintain employment.^46^ It can include computer literacy and money management skills to improve independence when employed and maintaining employment.^46^ However, Andre and Reddy^47^ mention that without the necessary funding and policy support for organisations that facilitate skills training that the long term success of these programmes are negatively affected.

The support from organizations in the form of shelter, safety, skills training, and rehabilitation programs, facilitate youth who are homeless to be employed. Shareen and Rio^40^ stated that the support, guidance, and assistance from role models can develop trustable, motivational and hopeful relationships.^39^ The support will promote belonging and provide an opportunity for the youth who are homeless to share their personal experiences, struggles and growth. Shareen and Rio^48^ showed that support being provided in the form of affordable housing could eventually end homelessness. If these psychosocial supports are not provided then it could ultimately affect an individual's employment status, thus affecting their health and work life balance^49,50^

## Conclusion

The purpose of the study was to explore and describe the experiences and perceptions of homeless youth with regard to the influence of job insecurity on their worker role. The study in particular tried to explore the barriers, facilitatory factors and how individuals who are homeless adapt to their worker roles. The findings of the study suggest that despite homeless youth's financial strain; they do experience challenges that influence their job insecurity. Furthermore, findings suggest various barriers limiting homeless youth from securing employment which include poor level of education and the influences of rebellious behaviour. Limited employment opportunities and stigma within the community towards the homeless youth also being viewed as additional barriers experienced. Despite the challenges these homeless youth face, several enablers aid in the job security and employment opportunities. The enablers in this study included programs facilitating community reintegration and programs which enhance participants’ occupations which significantly increases their worker role. It was found that skill enhancement, self-growth, and rehabilitation support significantly increases employment opportunities for homeless youth which does help the youth who are homeless to adapt to difficult circumstances.

Recommendations that surfaced from this study would be to facilitate the development of more structured vocational skills training programs for youth who are unemployed, that enables them to find employment in critical work related sectors such as information technology, infrastructure development and product manufacturing sectors. Enabling the youth to engage in training of specific skills that are needed to engage in the work sector such as basic budgeting skills and basic computer literacy.

## Limitations

A limitation experienced during this study was that most clients at the NGO is older than the sampling age (18–35 years old). Due to external circumstances many of the clients were unable to attend at the NGO during the week of interviews. The interviews were conducted across two different campuses of the NGO. The overarching limitation in the study was that the study sample consisted of six research participants (youth that were homeless) the majority of them being male. It is therefore suggested that future studies include a larger sample of research participants inclusive of more female participants. It may also be useful to possibly conduct focus groups with the research participants in addition to the semi structured interviews. Future studies include the above suggestions may contribute to greater generalizability of the study findings.

## Appendix 1: Semi-Structured Interview for Participants and Key informants:

Participants

1. Can you describe the challenges that you experienced in finding employment?
  - Probe: Please describe why you feel that… is or was a challenge.
  - Probe: Please describe what about your job was problematic.
  - Probe: Please describe the circumstances that caused you to struggle.
2. Can you describe the things that helped you maintain and find employment?
  - Probe: Please describe the circumstances that enabled you to keep your job or maintain your job.
  - Probe: Please describe the organisations or people that helped you find a job or maintain your job.
  - Probe: Please describe whether there is any specific training that helped you find a job or maintain your job.
3. Could you describe how you have managed to stay working despite being homeless?
  - Probe: How did you manage to cope with your job while being homeless?
  - Probe: How did you manage to return to work after being homeless?
4. If you had a magic wand or all the power in the world to develop a programme to help youth that are homeless find employment, how would you do this?
  - Probe: Please describe the main features of the programme.
  - Probe: What would a person need in order to make this programme work?
  - Probe: What would a person need to improve the work skills of youth who are homeless?

Key Informants:

1. Can you describe the challenges that you think youth who are homeless experience when finding and maintaining employment?
  - Please describe why you feel that… is or was a challenge.
  - Probe: Please describe what about their job you think is problematic.
2. Can you describe the things that helped youth who are unemployed find and maintain employment?
  - Probe: Please describe the circumstances that enabled them to keep their job or maintain a job.
  - Probe: Could you describe the organisations or people that helped them find a job or maintain their job?
  - Probe: What type of work-related training helped or would help them find a job or maintain their job?
3. Can you describe the patterns of individuals who are homeless in finding and keeping a job?
  - Probe: How do you think homeless people manage to return to work after becoming homeless?
4. If you had a magic wand or all the power in the world to develop a programme to help youth that are homeless find employment, how would you do this?
  - Probe: Please describe the main features of the programme.
  - Probe: What would a person need in order to make this programme work?
  - Probe: What would a person need to improve the work skills of youth who are homeless?
